# Balancing Prediction and Surprise: A Role for Active Sleep at the Dawn of Consciousness?

**DOI:** 10.3389/fnsys.2021.768762

**Published:** 2021-11-05

**Authors:** Matthew N. Van De Poll, Bruno van Swinderen

**Affiliations:** Queensland Brain Institute, The University of Queensland, Brisbane, QLD, Australia

**Keywords:** REM sleep, consciousness, predictive coding, emotions, invertebrate

## Abstract

The brain is a prediction machine. Yet the world is never entirely predictable, for any animal. Unexpected events are surprising, and this typically evokes prediction error signatures in mammalian brains. In humans such mismatched expectations are often associated with an emotional response as well, and emotional dysregulation can lead to cognitive disorders such as depression or schizophrenia. Emotional responses are understood to be important for memory consolidation, suggesting that positive or negative ‘valence’ cues more generally constitute an ancient mechanism designed to potently refine and generalize internal models of the world and thereby minimize prediction errors. On the other hand, abolishing error detection and surprise entirely (as could happen by generalization or habituation) is probably maladaptive, as this might undermine the very mechanism that brains use to become better prediction machines. This paradoxical view of brain function as an ongoing balance between prediction and surprise suggests a compelling approach to study and understand the evolution of consciousness in animals. In particular, this view may provide insight into the function and evolution of ‘active’ sleep. Here, we propose that active sleep – when animals are behaviorally asleep but their brain seems awake – is widespread beyond mammals and birds, and may have evolved as a mechanism for optimizing predictive processing in motile creatures confronted with constantly changing environments. To explore our hypothesis, we progress from humans to invertebrates, investigating how a potential role for rapid eye movement (REM) sleep in emotional regulation in humans could be re-examined as a conserved sleep function that co-evolved alongside selective attention to maintain an adaptive balance between prediction and surprise. This view of active sleep has some interesting implications for the evolution of subjective awareness and consciousness in animals.

## Introduction

Why do we dream? Every human since the dawn of humanity must have asked themselves this bewildering question, which seems inextricably linked to another related question: why do we sleep? It is therefore quite astounding to note that it was only about 100 years ago that a distinct sleep stage was identified – rapid eye movement (REM) sleep – that seemed to be associated with vivid dream reports (Loomis et al., 1937; Aserinsky and Kleitman, 1953), and that was different from other sleep stages such as slow-wave sleep (SWS; Blake and Gerard, 1937). Humans were probably always aware that other humans, or their animal companions, were engaging in different kinds of sleep. Their bed partners might twitch during their sleep sometimes or breathe deeply other times, their babies might suddenly smile, their dogs whined or padded the air with their paws (but only sometimes). These were all clues that different kinds of sleep were potentially at play, but it required the advent of brain recordings and electro-encephalography (EEG) in the last century to conclusively show, in humans as well as other mammals, that these were indeed distinct sleep stages. We now know that REM sleep is associated with wake-like electrical activity across the mammalian brain cortex, characterized by low-amplitude, desynchronized field potentials (Aserinsky and Kleitman, 1955; Jouvet, 1961; Hobson, 2009a). In contrast, with its unique high-amplitude slow waves (1–4 Hz ‘delta’ waves), SWS seemed different enough to wakefulness to have traditionally attracted more interest as somehow being ‘real’ or ‘deep’ sleep, potentially achieving some more crucial functions than REM sleep. Early on it was discovered that this distinct sleep stage, REM, was strongly associated with the subjective state of disconnected consciousness we term dreams, the often absurd or embarrassing nature of which did little to improve the standing of REM.

**FIGURE 1:**
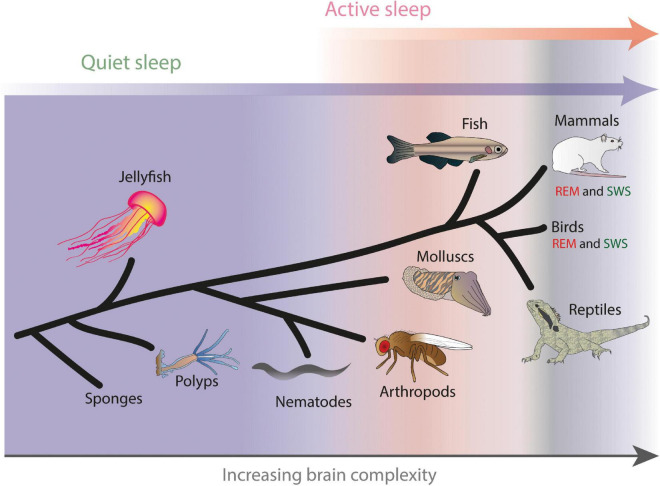
Hypothesized evolution of active and quiet sleep, with rapid eye movement (REM) sleep and slow wave sleep (SWS) in mammals and birds representing specialized solutions to achieving distinct sleep functions. Example animals where different forms of sleep have been characterized are shown, arranged schematically by increasing brain complexity. Adapted from Kirszenblat and van Swinderen (2015).

To date, almost every animal that has been investigated carefully (meaning, satisfying key behavioral criteria such as quiescence, increased arousal thresholds, and homeostatic regulation (Campbell and Tobler, 1984), has been found to need sleep. Beyond mammals and birds, this ranges from animals without central nervous systems (or ‘brains’) such as hydra (Kanaya et al., 2020) and jellyfish (Seymour et al., 2004; Nath et al., 2017), and roundworms (Raizen et al., 2008) to insects (Tobler and Neuner-Jehle, 1992; Shaw et al., 2000), fish (Zhdanova et al., 2001; Prober et al., 2006; Yokogawa et al., 2007), amphibians (Libourel and Herrel, 2016), and reptiles (Tauber et al., 1966; Ayala-Guerrero and Mexicano, 2008). All these animals become periodically quiescent (i.e., immobile) in order to engage important biological processes that are largely incompatible with waking activity and ongoing behavior. These processes include cell repair mechanisms, growth and development, waste and metabolite clearance, and stress regulation (Sassin et al., 1969; Xie et al., 2013; Ogawa and Otani, 2014; Tononi and Cirelli, 2014). In humans and other mammals, these basic cellular sleep functions typically occur during SWS, when the cortex is traversed by slow ‘delta’ waves (Dijk et al., 1990) but the rest of the brain is more quiet (Siegel, 2008). This suggests that ancient sleep functions important for maintaining neuronal health have been packaged into SWS in mammals and birds, and that the slow (1–4 Hz) waves characteristic of SWS in these animals are probably a thalamocortical novelty riding on a more ancient drive for periodic brain quiescence. All animals appear to need such periodic neural quiescence in order to develop and adapt appropriately to their environment. In contrast, only a subset of animals seem to engage in REM sleep (Figure 1).

During REM sleep, the brain looks awake but animals remain significantly less responsive to the outside world (Green and Arduini, 1954), so based on increased arousal thresholds alone this has qualified as ‘sleep’ (Andrillon and Kouider, 2020). Since this is potentially confusing (why are we then not awake and responsive?), REM sleep has also been termed ‘paradoxical sleep’ (Jouvet-Mounier et al., 1969) or ‘active sleep’ (Libourel and Herrel, 2016). The recent discovery of REM sleep-like sleep in disparate animals such as reptiles (Shein-Idelson et al., 2016), fish (Leung et al., 2019), and molluscs (Iglesias et al., 2019; Medeiros et al., 2021) casts doubt on a common evolutionary origin for REM sleep and instead suggests a selective pressure to achieve related ‘active sleep’ functions in these diverse creatures. What might these functions be? While ‘deep sleep’ functions seem easier to comprehend (i.e., recurrent neural quiescence is required for achieving cellular homeostasis), why should some animal brains remain wake-like but disconnected from the outside world? This seems a potentially hazardous prospect, with some cuttlefish for example engaging in striking chromatophore pattern displays during this purported sleep stage (Frank et al., 2012; Iglesias et al., 2019) – clearly not a good idea for an animal not paying attention to potential predators. REM sleep must therefore be performing an important function (or multiple functions), to offset the disadvantage of being disengaged from the immediate environment. That active as well as deep sleep stages might even be required for the smallest animal brains, such as flies (van Alphen et al., 2013; Yap et al., 2017; Tainton-Heap et al., 2021), argues for conserved functions linked to the evolution of central nervous systems, or brains.

In this hypothesis article, we review sleep across phylogeny and propose why some animal brains might need ‘active’ sleep, in addition to deep or ‘quiet’ sleep. We examine potential REM sleep functions based on the human and mammalian literature, and then work back from mammals to invertebrates to unpack these functions to some likely evolutionary antecedents. Our hypothesis is that active sleep provides a closed environment for optimizing attention-like processes centered on prediction, ensuring that the real world is predictable enough while maintaining a capacity for surprise. In humans, surprise is associated with emotions, and accordingly REM sleep in humans has been strongly associated with emotional regulation. We propose that this sleep stage has less to do with emotional regulation *per se* and more with an ancient animal need to balance prediction and surprise, in order to be optimally adaptive. We end with a discussion on how active sleep and consciousness might be linked in all animals that have a selective attention and are able to make predictions about what happens next.

## Part 1

### Rapid Eye Movement Sleep Is Active Sleep

#### Evidence From Humans

Some of the earliest accounts for sleep and dreaming describe it as either the result of a ‘cooling’ of the blood during the night or the wicking of an internal fire, while dreams are conjectured to be projections from the divine realm into mortals (Barbera, 2008). Perhaps these theories arose as a way to explain the enforced inactivity and unresponsiveness of sleep, as apart from an obvious continuation of breathing, during this state we appear to others as insensate and immobile. Indeed, this primordial view of sleep as the opposite of activity has led some thinkers to propose that its key function was to keep us safely quiescent in our caves or our trees while predators prowled during the night (Meddis, 1975).

However, over time a number of biological functions have been proposed for sleep, beyond simple inactivity. Before outlining these proposed sleep functions, we first briefly review some important observations about sleep architecture. In humans, a normal sleep cycle consists first of a fairly rapid transition from drowsiness into SWS (usually in the order of minutes). During SWS, the neuronal activity of the brain’s cortex is dominated by slow (1–4 Hz) oscillations (termed delta), which have been hypothesized to promote synaptic rescaling (Crunelli and Hughes, 2010; Tononi and Cirelli, 2014; de Vivo et al., 2017; Malafeev et al., 2018). Tellingly, the amplitude of delta activity is greatest at the start of sleep and decreases during successive bouts of SWS throughout the night (Dijk et al., 1990), and the amplitude of these delta waves has been reported to be proportional to the magnitude of sleep pressure experienced by the individual, suggesting homeostatic regulation of processes that accrued during sustained wakefulness (Dijk et al., 1990). That some of these processes involve accrued substances in the brain that need to be normalized after extended wakefulness seemed intuitively obvious; early findings revealed that the cerebrospinal fluid of sleep-deprived animals promotes sleep when injected into waking individuals (Ishimori, 1909; Legendre and Piéron, 1913). This suggested that sleep engages key molecular processes involved in cell health and development. Indeed, more recent studies have associated Non-REM sleep with cell growth and proliferation (Guzmán-marín et al., 2003; Sippel et al., 2020), DNA repair (Cirelli et al., 2004; Vyazovskiy and Harris, 2013; Zada et al., 2019), and waste clearance (Xie et al., 2013). Evidence for these basic cellular functions are supported by observable physical changes in the brain: during the SWS stage of Non-REM sleep, the interstitial space between neurons and glia expands, potentially allowing for more effective clearance of metabolites and other neuronal waste products via the glymphatic system (Xie et al., 2013; Jessen et al., 2015; Fultz et al., 2019). Additionally, glucose usage in the brain is far lower during SWS than during waking, implying that a resetting of local energy stores may be occurring during this sleep stage (Netchiporouk et al., 2001). Thus, the role of SWS in promoting homeostatic cellular processes in the brain is becoming increasingly understood, and it is intuitively obvious how these processes might have also been best deployed during sustained epochs of inactivity in the first animals.

The predominance of SWS in humans begins to fade typically an hour into sleep, to be replaced by periodic (∼90 min) alternations between SWS and REM (Malafeev et al., 2018). While delta wave amplitude decreases across successive SWS bouts, the duration of the REM bouts increases over sleep time. As with SWS, this may be indicative of homeostatic regulation. However, in contrast to SWS, the functions of REM have proved more difficult to uncover. Nevertheless, in humans REM has been associated with both consolidation of learning (Karni et al., 1994; Boyce et al., 2016) – a function it shares with SWS (Giuditta et al., 1995) – as well emotional regulation (Clemes and Dement, 1967).

REM sleep is also associated with distinct physiological signatures. During REM our constant regulation of internal body temperature (homeothermy) is suspended (Henane et al., 1977; Parmeggiani, 1990). Simultaneously, broad waves of activity originating in the brainstem sweep across the cortex (Jouvet, 1961; Hobson and Friston, 2012), our eyes twitch in their sockets (Aserinsky and Kleitman, 1955; Hong et al., 2009) and penile/clitoral erections are common (Fisher et al., 1965). These are all signs that the brain during REM is active, but in this case, it is internally generated or spontaneous activity rather than responses to the external sensorium. The traveling waves of neural activity (termed PGO waves, for their origin in the pontine-geniculo-occipital nuclei) in particular may resemble evoked visual-like activity in sensory cortices, stimulating wake-like activity (Andrillon et al., 2015; Andrillon and Kouider, 2020). Similarities with waking notwithstanding, REM sleep cannot be simply regarded as a waking state that happens to occur while we are asleep. For example, levels of inhibition in the visual system are lower during REM than wake (Lu et al., 2006; Hobson, 2009b) and the functional organization of the visual cortex and other areas of the brain is more locally confined than during wake (Wehrle et al., 2007). Finally, and perhaps most importantly, arousal thresholds are high during REM sleep (i.e., a strong stimulus is required to return the subject to the ‘real’ world), and can even be as high as during SWS (Ermis et al., 2010). This raises the question then: Why is a separate stage of sleep needed by the brain, one with wake-like levels of activity? And conversely, what is being accomplished during REM that requires the brain to be largely disconnected from sensory apparatuses?

In science generally an effective assessment of the necessity and functionality of a process is to observe what happens when it is removed or interrupted, although in the question of sleep it can be difficult to disambiguate effects of sleep loss from stress. In the past researchers have experimented with sustained sleep deprivation in humans, finding that perceptual distortions (i.e., hallucinations) were a common result, as well as mood changes and other deleterious cognitive effects (Dement, 1960; Waters et al., 2018). As early as the 1960s it was proposed that the appearance of daytime hallucinations as a result of sleep deprivation was REM-related, as an ‘intrusion’ of dreams into the waking world (Vogel, 1968). This may be due to the fact that beyond the physiological aspects detailed above, REM is the sleep stage most commonly associated with vivid dreaming (Aserinsky and Kleitman, 1953). While dreams do occur also during Non-REM states, they are typically much less ‘dreamlike’ and feature a significantly lower number of narrative events (Blagrove, 1993). In contrast, dreams during REM are typically emotionally charged and frequently play upon themes of anxiety or danger (Nielsen et al., 1992). Interestingly, the level of emotional content present within dreams occurring during either early REM or late REM may be predictive of successful emotional regulation (Cartwright et al., 1998). However, for the purposes of this review it should be noted that we are not interested in analyzing dream content, but rather in why this sleep stage should be needed at all. The ‘dream pressure’ hypothesis (reviewed in Berger and Riemann, 1993) proposes that emotional (and particularly, negative) events generate a ‘pressure’ to dream that decreases the latency to REM sleep. This may be mechanistically similar to the relationship between sleep pressure and the greater amplitude of early delta waves in SWS, but for emotional content. The implication here is that daytime neurological activity creates a need for cellular homeostatic processes, which are fulfilled by proportional increases in delta activity during SWS, while daytime cognitive or emotional events generate a need for homeostatic regulation by REM sleep. With this hypothesis in mind, we next examine the evidence for active sleep in animals beyond mammals and birds (where they have already been well documented and reviewed (e.g., see Miyazaki et al., 2017; Lesku et al., 2019).

### Active Sleep in Other Animals

#### Active Sleep in Reptiles and Fish

Much like originally in humans, sleep in reptiles and fish has previously been viewed as a simple down-state of decreased brain activity (Siegel, 2008; Libourel and Herrel, 2016), without the delta waves characteristic of mammalian sleep, and without the active paralysis and twitches characteristic of REM. Not surprisingly, this premature conclusion may have been more due to absence of evidence rather than evidence of absence. Importantly, the key neural signatures for identifying sleep stages in mammals are biased toward animals that have a well-developed cortex, the specialized brain tissue capable of generating the kinds of electrical fields that EEG electrodes are designed to detect. This neo-cortical definition of sleep often ignores the rest of the brain, which is largely inaccessible to electrodes placed on the skull’s surface. As discussed above, deeper brain recordings into the brainstem of cats and rodents revealed volleys of activity (PGO waves) associated with REM sleep (Jouvet, 1961; Kaufman and Morrison, 1981), suggesting that this more ancient ‘reptilian’ part of the brain is involved in regulating REM sleep function. It may therefore not have been surprising to discover that reptiles also appear to display a REM-like sleep stage, which alternates with a form of SWS (Libourel and Herrel, 2016; Shein-Idelson et al., 2016). To identify these distinct sleep stages in Australian central bearded dragons (*Pogona vitticeps*), the authors relied on intracranial recordings coupled to filming the reptiles’ microbehaviors, such as their eye movements. Instead of specifically identifying neural oscillations such as delta, the authors quantified an ongoing ratio between high and low frequency domains during sleep and correlated these to the animal’s physiology and arousal thresholds. Interestingly, the authors found that bearded dragons appeared to cycle rapidly between sleep stages, with a periodicity of about 80 s (Shein-Idelson et al., 2016). Having identified distinct sleep stages in reptiles, there has so far been little further work in understanding why a lizard might need REM sleep. Examining cognitive functions in lizards is not obvious, as there are few reliable behavioral learning paradigms available.

Fish have been a relative latecomer to sleep research, likely due to the fact that it is difficult to secure electrodes and record electrical activity from unrestrained underwater creatures (but see Ramón et al., 2004; Dunlop and Laming, 2005), coupled with the reliance on brain activity as a readout for sleep. However, with the advent of new techniques researchers in this area have rapidly made up for lost time by exploiting the power of one species in particular, the genetic model *Danio rerio*, or common zebrafish. Following some early observations that freely swimming zebrafish do indeed need to sleep (Zhdanova et al., 2001; Prober et al., 2006; Yokogawa et al., 2007), a breakthrough in assessing neural correlates of sleep in these animals came by exploiting genetically encoded calcium sensors (Chen et al., 2013) expressed in their brain. In a recent study, Leung et al. (2019) imaged the activity of neurons across the brains of sleep-deprived zebrafish that were then restrained for imaging calcium as well as a suite of polysomnography readouts under a microscope. The authors found what appeared to be two distinct forms of brain activity: a putative ‘quiet’ sleep stage and an ‘active’ sleep stage (Leung et al., 2019). The former displayed synchronized activity in only a small subset of cells, with most of the rest of the brain becoming quiet. In contrast, active sleep was characterized by volleys of neural activity within the dorsal pallidum, and associated with a number of other physiological readouts (e.g., irregular heartbeat and loss of muscle tone) reminiscent of REM sleep in mammals and birds, but without any rapid eye movements. Together with the earlier behavioral work in this model, these studies support the idea that active sleep has deeper evolutionary roots (and, hence, likely functions) than the mammal-bird-reptile lineage. Importantly, this evidence from zebrafish has spurred the field to move away from neocortical identifiers of sleep stages (e.g., slow-wave sleep and REM sleep) to their likely evolutionary antecedents: quiet sleep and active sleep (Figure 1). We therefore next examine evidence for these distinct sleep stages even further down the evolutionary tree, in invertebrates.

#### Active Sleep in Invertebrates

In our search for distinct sleep stages among invertebrates, it may seem logical to begin with what would superficially appear to be the ‘smarter’ ones, such as octopi and honeybees. Octopi can plan ahead (Finn et al., 2009), bees can learn context and abstract concepts (Giurfa, 2007), and both use their bodies to communicate complex information with conspecifics (von Frisch, 1967; Young, 1991). Changes in body pigmentation are also evident in relatives of octopuses, such as cuttlefish, and these rapid changes in colors and patterns have also been tentatively associated with emotional states in these animals (Young, 1991; Scheel et al., 2016). Recent work examining sleep in cuttlefish found a behavioral state where the cephalopods were clearly asleep (quiescent and unresponsive) while their pigmentation rapidly flashed a variety of changing patterns, in contrast to other quiescent states where this did not occur (Frank et al., 2012). Without brain recordings, it remained uncertain if this is indeed a form of active sleep, but this has now been confirmed with electrophysiological evidence in a more recent cuttlefish sleep study (Iglesias et al., 2019), as well as in behavioral evidence from octopuses (Medeiros et al., 2021). Importantly, in this last octopus study, careful examination of other microbehaviors allowed the authors to determine transition probabilities between these different sleep states and wakefulness, and these findings further confirm the existence of a complex sleep architecture in invertebrate brains.

Early evidence that sleep architecture might be complex in honeybees relied primarily on filming their microbehaviors in the hive, where they rested. There, it was observed that honeybee antennae moved in a regular, circular pattern soon after sleep onset, and this movement diminished toward the middle of a sleep bout (Sauer et al., 2003), after which the honeybee body lay closer to the substrate, with their antennae drooping and mandibles resting on the surface (Kaiser, 1988). More recent research has confirmed these observations, and shown that bees indeed have deeper and lighter sleep stages linked to changes in microbehaviors (Klein et al., 2014; Zwaka et al., 2015). However, again the absence of electrophysiology (or any other kind of brain recording) makes it difficult to confirm whether these indeed represent ‘active’ and ‘quiet’ sleep, as has been documented in vertebrates (but see Kaiser and Steiner-Kaiser, 1983, for evidence of loss of neural responsiveness in sleeping honeybees).

There has been some sleep electrophysiology work done in one unlikely invertebrate, the Louisiana crayfish. In a series of studies performed initially in collaboration with the renowned electrophysiologist Ted Bullock (who recorded from many invertebrates; see Zupanc, 2006), Mexican neuroscientist Fidel Ramón described ‘slow’ (∼5–10 Hz) oscillatory signatures in the central brain of sleeping crayfish (Ramón et al., 2004). During this sleep stage, crayfish often adopted a stereotypical posture, lying on their side. Subsequent studies from the same group examining these sleep signatures more carefully concluded that local field potential (LFP) activity in sleeping crayfish was not ‘slow,’ but closer to the beta or low gamma range (15–30 Hz) (Mendoza-Angeles et al., 2010, 2007). Whether this brain activity is always present in sleeping crayfish is unclear, although the authors note that crayfish could adopt other sleeping positions, such as ‘crouched’ (Mendoza-Angeles et al., 2007). Postural differences during sleep may suggest a form of sleep paralysis, for example the sideways position associated with 15–30 Hz brain activity, but it remains unclear if this is active sleep. As we know from SWS in mammals, neural oscillations do not necessarily indicate wake-like brain activity, which should ideally be verified by neural firing rates. It is nevertheless evident from this work that the arthropod brain does not necessarily become more quiet during sleep, and that sleep-related oscillations seem to emanate from a part of the central arthropod brain termed the ‘central complex’ (Mendoza-Angeles et al., 2010).

#### Active and Quiet Sleep in Fruit Flies

The fruit fly, *Drosophila melanogaster*, occupies a special place in sleep research because so much more work has been done on sleep in this model organism over the past two decades, compared to other invertebrates. Sleep was originally identified in *Drosophila* by using re-purposed circadian activity monitors, wherein walking flies interrupting an infrared beam reveal their locomotor activity levels over successive days and nights. Five minutes or more without any beam-crossing was found to be associated with higher arousal thresholds, and thus by inference, sleep (Hendricks et al., 2000; Shaw et al., 2000), and this 5-min criterion was then used for almost all subsequent sleep research in this model, with a view to unraveling the cellular and molecular underpinnings of sleep physiology and function in a simple and genetically tractable model (see Cirelli, 2009; Ly et al., 2018 for recent reviews). This logic held as long as sleep was considered a single state in flies, with a common underlying set of mechanisms and functions. Behavioral experiments probing arousal thresholds more carefully showed that this assumption is unlikely to be true: flies display changing, often cycling, levels of behavioral responsiveness across a sleep bout – deeper sleep and lighter sleep (van Alphen et al., 2013). Further, daytime sleep is significantly lighter than night-time sleep, supporting earlier observations that sleep duration architecture varies between day and night in flies (Ishimoto et al., 2012). More recent behavioral studies using continuous video tracking instead of infrared beams also support the suspicion that flies sleep in different lighter and deeper stages (Wiggin et al., 2020; French et al., 2021; Xu et al., 2021). The realization that sleep might be just as complex in this smallest of animal brains as in higher organisms raises some questions regarding the wealth of correlational data gathered in this sleep model over the past two decades. Indeed, a bewildering variety of neural structures and proteins have been found to be associated with fly sleep (see, for example Dubowy and Sehgal, 2017), if sleep is considered to be a single state based upon a 5-min inactivity criterion. It is now evident that these various structures and proteins probably encompass distinct sleep stages and thus functions, which may have been confounded together. As an analogy, if SWS and REM were confounded in mammals, we would be calling almost every neurotransmitter from acetylcholine to GABA as sleep-relevant and grouping varied structures all the way from the brainstem to the cortex as regulating the same phenomenon, which would obviously be misguided.

Evidence for different sleep stages in *Drosophila* was affirmed with brain recordings in tethered flies walking (or sleeping) on an air-supported ball. The first evidence was electrophysiological, where LFPs recorded from the brains of spontaneously sleeping flies revealed patterns of distinct oscillatory activity alternating with overall decreased LFP activity (Yap et al., 2017). Interestingly, the oscillatory LFP activity was observed to be in the 7–10 Hz frequency range (‘theta’ band), and was found to emanate from the vicinity of the central complex (Yap et al., 2017; Troup et al., 2018), which aligns with earlier observations from sleeping crayfish – described above. In contrast, ‘deep’ sleep in flies did not appear to be associated with any specific oscillatory activity, just decreased overall LFP amplitudes (but see Raccuglia et al., 2019 for evidence of ‘delta-like’ synchronization of neural firing in the central complex of sleep-deprived flies).

Additional support for the idea that flies sleep in distinct active and quiet stages has come from calcium imaging, the same genetic strategy used to identify these distinct stages in sleeping zebrafish. Here, tethered flies placed on an air-supported ball slept spontaneously while a 100 μM volume of neurons in their central brain was imaged using 2-photon microscopy (Tainton-Heap et al., 2021). Tracking the activity of thousands of neurons this way, in waking and sleeping flies, confirmed the complexity previously seen with electrophysiology: brain activity remained wake-like well into the first 5 min of sleep, then decreased to lower levels, and then could increase again to wake-like levels even in flies that remained immobile throughout. Importantly, by tracking the identities of individual neurons throughout a sleep bout, the authors showed that successive active and quiet sleep stages engaged largely non-overlapping groups of cells, suggesting different circuits were recruited and potentially different functions were being served. Indeed, there is now good evidence that active sleep in flies engages a structure in the central brain called the fan-shaped body, which has been linked to sensory processing (Hu et al., 2018; Sareen et al., 2020) and visual learning and attention (Liu et al., 2006; de Bivort and van Swinderen, 2016; Tainton-Heap et al., 2021). In contrast, quiet sleep in flies may be more important for basic cellular homeostatic processes, such as waste clearance (van Alphen et al., 2021) and repair (Stanhope et al., 2020; Bedont et al., 2021). In this way, active and quiet sleep functions in flies may align logically with some proposed REM and SWS functions in mammals, as outlined above.

One conclusion from the admittedly narrow slice of work done in invertebrates suggests that all animals endowed with a brain might sleep in distinct stages, which we propose are best described as active and quiet sleep, and these stages share functional properties with REM and SWS respectively in mammals and birds (Figure 1). But what of invertebrates that do not have a brain (or a proper central nervous system), such as sponges, polyps, jellyfish, or certain roundworms? With these, it is possible that only a quiet sleep stage might be operating, tightly linked to periodic developmental or other cell-homeostatic needs (Raizen et al., 2008; Nath et al., 2017; Kanaya et al., 2020). The roundworm *Caenorhabditis elegans* becomes transiently quiescent when growing out of different larval stages (Raizen et al., 2008) or following periods of acute stress (Hill et al., 2014), but there is no evidence (yet) of wake-like levels of neural activity in a quiescent, completely immobile nematode. It is important here to consider recording preparations: calcium imaging in animal models typically requires immobilization of the preparation. While fly or fish brains immobilized under the microscope can co-exist with attached moving legs or tails (to verify sleep or increased arousal thresholds), immobilized nematodes are just that: a worm in plastic straitjacket, unable to move at all. Reports of ‘brain’ activity during ‘sleep’ in such preparations (Nichols et al., 2017) should therefore be interpreted with caution, although it remains possible that even these simple animals require periods of active sleep, in addition to quiet sleep. A case could nevertheless be made for why some animals might not need active sleep, and why all animals might need quiet sleep: not all animals are endowed with a capacity for selective attention (Kirszenblat and van Swinderen, 2015). This debate returns us to our early discussions disambiguating possible REM and SWS sleep functions in humans, with a view to then exploring how some of these functions may have already been required in simpler animals engaged in active sleep.

## Part 2

### A Role for Rapid Eye Movement Sleep in Emotional Regulation

Having postulated earlier a connection between the wake-like state of REM and emotional regulation in humans, we will now review some evidence for this linkage. We start by discussing the emotion-related effects of altered levels of REM and then move on to ties between REM and common psychological pathologies. In addition, we will briefly review evidence from other mammals where the links between emotional regulation and REM sleep have been modeled and investigated. We then consider how this link might be modeled in invertebrates that display evidence for active sleep.

In insomniacs, REM fragmentation has been linked to emotional dysregulation and an inability to effectively process emotional stimuli (Galbiati et al., 2020) and thus, one attractive option for investigating the functions of REM is to observe the effect of its removal in normal and pathological subjects. With the advent of polysomnography and online analysis of EEG data, it has become increasingly tractable to accurately identify waking and sleeping states of experimental participants and to use this information to selectively interrupt specific sleep stages. For REM in particular, numerous studies have shown that it is involved in recall of emotional content (Nishida et al., 2009; Rosales-Lagarde et al., 2012; Wiesner et al., 2015). In the work of Rosales-Lagarde et al. (2012) it was shown that human participants deprived of REM were less able to accurately distinguish between trained and novel images containing negative emotional content but were unimpaired in their recall of emotionally neutral stimuli (Rosales-Lagarde et al., 2012). Wiesner et al. (2015) also utilized selective deprivation of both SWS and REM, showing that emotional memory consolidation (quantified as successful recognition of stimuli on the following day) was impaired by REM deprivation but emotional reactivity (self-reported on a survey) was unchanged between the deprivation groups (Wiesner et al., 2015). The implication here is that SWS may contribute to the regulation of emotional reactivity, while emotional consolidation is primarily controlled by REM.

A role for REM sleep in emotional memory consolidation can also be found through fear conditioning studies, as an alternative means to access emotion. Spoormaker et al. (2012) conditioned human subjects (with mild electric shocks) to feel fear toward simple visual shapes. These subjects then underwent extinction training (presentation of the shapes in an absence of the aversive shocks) after fear conditioning and were split into either a REM deprivation group or a group with a matched amount of Non-REM deprivation. It was found that REM deprivation significantly impaired the effectiveness of the extinction training, with REM deprivation participants exhibiting responses to conditioned stimuli closer to original than post-extinction levels (Spoormaker et al., 2012). This shows that REM sleep plays a key role not only in forming associations between emotional events and their eliciting stimuli but also in the weakening of such ties when applicable. In humans and other mammals, processing of emotional events during REM is proposed to revolve around activity in the amygdala and anterior cingulate cortex, such that impairment in normal functioning prevents effective emotional consolidation (Braun et al., 1997).

In rodent models of fear conditioning, it has been possible to begin investigating more carefully the links between REM and emotional learning. One reliable technique for selective REM deprivation in rodents involves a semi-submerged sleeping platform where Non-REM sleep (which does not require muscle relaxation) can be achieved but REM onset leads to sudden immersion and awakening (Arthaud et al., 2015). Early behavioral work using this approach showed that rats deprived of REM sleep had decreased acetylcholine levels (Bowers et al., 1966) and were more prone to fight following an unexpected foot shock (Morden et al., 1968). Conversely, it has been shown in mice that fear conditioning can lead to an increase in REM sleep in subsequent rest periods (Smith, 1985). In more recent rodent work it has been shown that muscarinic cholinergic receptors are critical for REM sleep (Niwa et al., 2018) and knockdown of cholinergic receptors significantly impairs fear conditioning, as well as other forms of learning (Queiroz et al., 2013). However, acetylcholine regulates a wide range of waking brain functions, so it is difficult to draw any strong conclusions between learning and REM sleep without considering other consequences of chronically downregulating cholinergic receptors in the mammalian brain.

One aspect through which ties between REM and emotional consolidation become salient is that of pathological symptomologies. In particular, the association between REM and depression is arguably the most classic neuropathology of negative affect (Berger and Riemann, 1993). Sleep studies with clinically depressed subjects have been performed since the 1940s (Diaz-Guerrero et al., 1946) and in these studies and the decades since it has been shown that depressed individuals tend to have reduced volume of SWS and shortened latency to enter REM sleep (Berger and Riemann, 1993). However, Vogel et al. (1980) showed that the total volume of REM was not significantly different between depressives and neurotypical individuals, and the change in REM architecture was primarily a shift toward ‘early REM’ in afflicted individuals. In more recent work, Harrington et al. (2018) recruited participants with minor and severe depression, finding that the degree to which participants consolidated new negative memories during a night of sleep was correlated with the severity of their depression and an increase in REM density. Notably, while there is evidence that REM deprivation leads to emotional instability (Clemes and Dement, 1967), there is also significant evidence supporting the use of selective deprivation of REM as a tool leading to improved outcomes for sufferers of depression (Vogel et al., 1980), although more recent evidence has cast doubt upon the REM specificity of this improvement (Giedke and Schwärzler, 2002). Many commonly prescribed antidepressants [such as the older tricyclic and tetracyclic antidepressants as well as more modern selective serotonin reuptake inhibitors (SSRIs)] have a REM-suppressing effect (Reyes et al., 1983; Riemann et al., 1990). One explanation for these seemingly contradictory findings is that both too much or too little REM is deleterious to normal emotional functioning, so these REM-suppressing antidepressants may improve depression by returning the latency of REM onset to a normal point (Reyes et al., 1983). There have also been other propositions for the mechanism behind emotional improvements following REM deprivation, ranging from a resetting of a biological oscillator (Vogel et al., 1980), to prevention of dreams containing negative emotional content during early REM epochs (Cartwright et al., 1998).

However, changes in REM quantity are not just associated with depression; other neurological disorders including post-traumatic stress disorder (PTSD) (Yetkin et al., 2010), obsessive-compulsive disorders (OCD) or eating disorders (Berger and Riemann, 1993) and schizophrenia (Zarcone et al., 1987) have all been linked to alterations in this sleep stage. But why might REM be increased in patients with these diseases in the first place? One possibility is that depression, PTSD, schizophrenia, OCD and other cognitive disorders are different manifestations of similar underlying neuropsychological issues (Plana-Ripoll et al., 2019; Hobson et al., 2021), or alternatively that the dysregulation of emotional content invariably involves a REM element. This “chicken or egg?” question is centralized around whether it is the disorders that lead to dysregulated REM sleep, the dysregulated sleep that leads to disorders, or a combination of both possibilities. The difficulty of determining whether altered REM architecture is a cause or consequence of cognitive and emotional disorders calls for a reductionist approach where key aspects of REM sleep, such as emotional regulation, might be modeled. Although there is no evidence that REM sleep evolved from invertebrate active sleep, the discovery of active sleep in a variety of simpler animal models provides a way forward for understanding potentially conserved sleep functions. However, with the evidence from humans and rodents pointing so strongly toward emotional regulation, how can this even be modeled in animals such as flies?

### Emotions in Arthropods?

There have been a few published efforts to determine whether arthropods display emotional responses. Although emotions seem to be largely subjective, thus opaque to anyone beyond ourselves, they also betray a short list of clearly measurable correlates which can be used as potential evidence. These correlates are centered around measures of arousal or bodily excitation, as well as evidence of valence, which can lead to attraction or repulsion to a stimulus. To uncover any evidence of a persisting ‘internal’ state, behavioral responses are then often dissociated from immediate stimulus parameters. For example, positive or negative valence might generalize to related stimuli or graded variations of the stimulus, or altered arousal states might persist well after the stimulus has disappeared (Anderson, 2016). Such criteria have been useful for studying aggression in a wide variety of arthropods, from crayfish to flies (Kravitz and Huber, 2003). Lean explanations of emotions however might view aggression as an innate response, much like phototaxis or courtship. To probe more deeply into learned emotional responses (e.g., something innate might be overturned after learning new associations), researchers have traditionally resorted to classical conditioning paradigms, by punishing or rewarding animals and then designing elegant experiments to see if some of the emotion-relevant criteria (e.g., scalability, persistence, generalization) are satisfied (Anderson, 2016). Typically, these experiments are designed to determine if animals are behaving ‘optimistically’ or ‘pessimistically’ when confronted with ambiguous stimuli, after positive or negative re-enforcement. For example, crayfish (*Procambarus clarki*, the same species discussed earlier) was found to display anxiety-like behavior after punishment (Fossat et al., 2014). Remarkably, this behavior could be corrected with the anti-anxiety medication chlordiazepoxide, developed originally for humans (Fischer et al., 2006). Similar experiments on honeybees showed the same result, with these clever insects displaying a form of pessimism about ambiguously colored flowers after being shaken (Bateson et al., 2011). Conversely (in a different study), when bumblebees received an unexpected reward immediately prior to performing a feeding choice task they were more likely to display ‘optimistic’ behavior by promptly moving toward ambiguous stimuli that control bees were slower to attend to Solvi et al. (2016). To gain traction, these behavioral studies often support their conclusions with pharmacological interventions, typically centered on drugs targeting monoaminergic systems such as dopamine and serotonin, which also regulate emotional responses in mammals (Anderson, 2016).

Any neuroethologist attempting to uncover evidence for emotions in insects is, however, confronted with a conundrum: we could in principle document bumblebees sobbing in grief at the death of a conspecific, tiny handkerchiefs and all, and a counterargument could always be made that this is nothing more than a series of innate behaviors, not emotion. This potential stalemate has led some in the field to take a different tack, that is to use reductionistic models such as *Drosophila* to simply better understand the neural underpinnings of arousal and the brain circuits regulating the variety of behaviors that might provide mechanistic evidence for scalability, persistence, and generalization. Thus, one *Drosophila* study (Gibson et al., 2015) designed a visual threat paradigm to measure defensive arousal in flies (‘fear’), hinting at the existence of dynamic internal states. Other studies have uncovered evidence of efference copy mechanisms (Blakemore et al., 2000) in the fly brain, suggesting that internal states (or motivations) gate the responsiveness of sensory neurons (Kim et al., 2015; Fujiwara et al., 2017). A recent study provides some additional convincing evidence for internal states in flies, by probing how visual processing might be dynamically gated by sexual arousal (Sten et al., 2021). If sexual arousal gates visual processing in flies, it seems likely that fear or anxiety might too, although there has not been much work done in unraveling the neural circuitry of fear in flies. In contrast, there has been much circuit-level work done on aggression (Hoopfer, 2016) and escape responses (Card and Dickinson, 2008; Fotowat et al., 2009), without any need to invoke emotions like anger and fear. This brings us back again to our original conundrum of how to disambiguate emotions from innate responses in these simpler models, and more specifically how to disentangle our anthropocentric views of emotion from their likely evolutionary antecedents. One way to proceed in this regard, and also to potentially better understand conserved functions being engaged by active sleep, is to study selective attention mechanisms and to consider how emotions are linked to predictive systems in the brain.

Like humans and rodents, insects pay attention to novelty. This means that, when confronted with novel objects [in a virtual reality environment, for example (Heisenberg and Wolf, 1984)], flies will orient toward objects they haven’t seen before and ignore competing objects they may be more familiar with (Dill and Heisenberg, 1995; Solanki et al., 2015). Interestingly, responses to visual novelty in flies can override innate visual preferences, meaning that flies will transiently fixate on innately ‘repulsive’ objects (e.g., a green square) over ‘attractive’ objects (e.g., a vertical green bar) if the otherwise repulsive object is novel (Grabowska et al., 2018). Earlier electrophysiological recordings from behaving flies showed that visual novelty is associated with transient oscillations in their central brain, in the range of 20–30 Hz (van Swinderen and Greenspan, 2003; van Swinderen, 2007). A more recent study recording directly from the central complex of behaving flies revealed a selective phase-locking mechanism between the endogenous 20–30 Hz oscillations and the attended object (Grabowska et al., 2020). This suggests a conserved mechanism in the fly brain attuned to first detecting surprising stimuli (i.e., novelty), and then to paying attention to them for a period of time (Sareen et al., 2011; van Swinderen, 2011). Interestingly, when an arousal system in the fly (neuropeptide F) is transiently activated, this increases 20–30 Hz phase locking in the fly brain and redirects the insect’s attention to novel objects irrespective of their innate valence (Grabowska et al., 2020). Such findings again suggest an evolutionarily conserved link between arousal systems and novelty detection mechanisms. To further consider this link with predictive mechanisms in the brain, and how they might be regulated by active sleep, we return to humans.

## Part 3

### Emotions Are Linked to Prediction Errors

There is an obvious purpose to emotions, which is to alert us about the consequences of our predictions. Unfulfilled predictions are jarring; we might feel sadness or anger when our favorite sporting team unexpectedly loses a match, or more acutely when we miss a goal kick. Similarly, there is a simpler satisfaction when a prediction is confirmed (for better or worse). In this way, emotions are a way to inform us that a salient event that failed to match our predictions has occurred, and that the circumstances that lead to this should be corrected and committed to memory. Numerous psychological studies have shown a relationship between the strength of emotional responses and the degree to which events were surprising (e.g., Feather, 1967; Bhatia et al., 2019). Notably, emotions seem to arise more from the deviation of expectation of an event rather than the magnitude of the event itself. In work by Villano et al. (2020), it was shown that for university students receiving their end of semester course marks, the strength of emotional affect experienced by the students was more strongly proportional to the deviation from their expectations than the mark itself. Additionally, and perhaps unsurprisingly, negative affect (resulting from lower than expected marks) was more profound than positive (Villano et al., 2020). These examples of high-level cognitive predictions are what we typically think of when associating emotions to surprising events. However, predictions can also reflect low-level (non-explicit) expectations, and these can also trigger emotional responses that might be rationalized afterward.

Recent theories seek to explain emotions as a way to understand both explained and unexplained deviations in our own internal state (Seth, 2013; Barrett et al., 2016). For example, Schachter and Singer showed in 1963 that participants who were administered an injection to increase their physiological arousal (in this case, a low dose of epinephrine), but not informed as to the effects of said injection, were more prone to sympathetic emotional influence from a conspirator who had been schooled to act in a particular emotional manner (Schachter and Singer, 1963). The implication here is that in the absence of their internal narrative providing them an obvious cause for their self-detected state of arousal, participants attributed their internal state as the result of a presumed emotional reaction. Similar evidence can be found in the classic psychological quirk of mood improvement following a pen being held in one’s mouth to artificially induce a smile (Labroo et al., 2014). Experiments such as these could be seen as attempts at divorcing emotional responses from the conscious states typically associated with them in humans, to potentially achieve a better understanding of their fundamental functions. One of these potential functions is to highlight that something unexpected has just occurred, which introduces predictive coding theory to our discussion.

**FIGURE 2:**
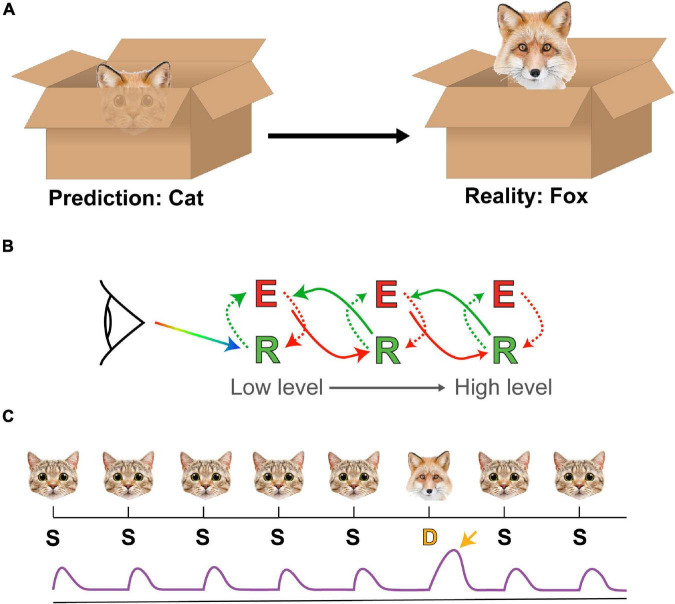
Predictive coding and oddball paradigms. **(A)** A prediction of the animal hiding in a box (a cat) based on a set of ears turns out to be in error (it’s a fox) when further details about the animal are revealed. In this case the prediction error is the misattribution of the animal as a cat. **(B)** A simple schema of core tenets of predictive coding theory. Sensory input (rainbow arrow) interfaces with a low-level representation (R) unit, which generates a mismatch that is used to refine an error (E) signal within a feedback architecture. This error signal also receives predictions from higher-level representation units while simultaneously supplying these units with updates. By arranging these units in a hierarchical manner, each layer can be used to represent different levels in processing, all the way from simple visual features such as orientation up to abstract concepts and ideas. **(C)** A schema of a simple oddball paradigm and prediction error signal. In this case an image of a cat (the Standard, S) is presented repeatedly, occasionally replaced with an image of a fox (the Deviant, D). The standards (S) evoke a reproducible response from the brain (purple trace) while the deviant (D) (typically matched for low-level features) evokes a different response (yellow arrow), which is detectable by EEG and/or functional magnetic resonance imaging (fMRI).

Predictive coding theory (Rao and Ballard, 1999) provides a compelling framework on which to better understand the importance of emotional regulation, based on notions of ‘unconscious inference’ first proposed by Hermann von Helmholtz (Helmholtz, 1860; Shipp, 2016). Predictive coding describes a system whereby sensory information about the world is used to generate an internal model that informs a system about the likely causes of said sensory stimuli. Sensory returns not matching this model represent prediction errors and the system can react to these by updating its model to better fit the evidence or by enacting change to bring the world into line with the model (Figures 2A,B). For these models to remain efficient and parsimonious, it is necessary for them to be regularly reviewed and reorganized, which is a role some have proposed for REM sleep (Hobson et al., 2014; Llewellyn, 2016; Windt, 2018).

In humans, predictive processing is commonly studied through the optics of ‘oddball’ paradigms, wherein a sequence of ‘standard’ stimuli is interrupted infrequently by a ‘deviant’ stimulus (Friston, 2005; Figure 2C). Under normal conditions this deviant stimulus elicits a prediction error signal in EEG recordings, visible in humans as a Mismatch Negativity (MMN), which is a distinct electrophysiological correlate of surprise (Näätänen, 1990). The usefulness of oddball paradigms lie in their versatility; virtually any sensory modality can be used for delivering stimuli and the semantic separation between standard and deviant stimuli can be as simple as “square vs. circle” (Huettel and McCarthy, 2004) or as complex as “repeated human face vs. novel human face” (Feuerriegel et al., 2018).

### The Sleeping Brain Makes Predictions

Notably, the human brain appears capable of generating certain prediction error signals even during the various stages of sleep. Previous studies have shown that human participants elicit electrophysiological markers of surprise in response to deviant stimuli during waking, Non-REM and REM sleep (Bastuji et al., 2002) and at least one study has shown that the rate of K-complexes during sleep may be tied to the salience of presented stimuli (Oswald et al., 1960). Notably, high-level signals of prediction error such as the P300 wave [so named because it is elicited around 300 ms after recognition of a deviation (Picton, 1992)] do not occur in response to oddball events during either Non-REM or REM sleep, but local detections of mismatch are present (Strauss et al., 2015). These prediction error signals have also been studied in the context of altered brain states such as general anesthesia (Koelsch et al., 2006) and coma (Bekinschtein et al., 2009), wherein the local mismatch response is typically preserved whereas more ‘conscious’ indicators of deviation fail to arise. Recognition of one’s own name, which has been long known to occupy a privileged space in human stimulus processing (Carmody and Lewis, 2006) is present even in sleep (McDonald et al., 1975), implying that it is a representation that may span all the way to the lowest levels of the auditory system. Thus, while the sleeping brain still appears to be able to categorize external events as surprising or not surprising, it remains unclear to what level different sleep stages regulate this important capacity of the brain.

Studies have shown that sleep in general seems important to the formation of predictive models (Wagner et al., 2004; Lutz et al., 2018). For example, improvements in prediction-associated performance were found by Wagner et al. (2004) on a digit transformation task with a hidden abstract rule. Under normal conditions participants would derive an answer for each task block by stepwise calculations, but it was also possible to infer the correct answer midway through each block if participants were to discover the hidden rule governing the digits, the existence of which was not communicated to participants. Comparing participants who were allowed an 8-h sleep against those who remained awake revealed that sleepers had a more than doubled likelihood of uncovering the hidden rule the following day, compared to participants who were awake for the same span (Wagner et al., 2004).

### Does Rapid Eye Movement Sleep Specifically Regulate Predictions?

Understanding that emotions provide a potential mechanism to recognize and correct prediction errors, and that REM sleep is involved in emotional regulation, immediately suggests that REM sleep might also be important for regulating prediction. Thus, we posit that rather than regulating emotions *per se*, REM sleep in fact regulates the *predictions* that drive our (human) emotional responses. Importantly, this view allows us to sidestep anthropocentric concerns on whether animals have emotions or not; they all make predictions.

The evidence for REM involvement in consolidation of learned tasks is extensive, but arguably the end goal of a consolidated model is for it to be actively used in a predictive capacity and, so far, human experimental literature directly linking together REM sleep specifically with predictive capacity remains relatively unexplored. Barsky et al., 2015 tested participants unconsciously learning to predict the ‘weather’ from hidden association probabilities with abstract stimuli before and after a nap. They found that the nap significantly improved participant’s ability to correctly guess the weather, and that REM quantity was correlated with success (Barsky et al., 2015). Earlier work by Cai et al. (2009) showed that REM sleep specifically was important for improvement in creative problem solving, involving recombination of learned sequences with an unrelated cognitive task. Notably, the brain is capable of forming models of stimulus properties even without conscious direction (Barbosa and Kouider, 2018), which is probably one component underlying the means by which a sleep state like REM can have such a seemingly cognitive role. For paradigms targeting unconscious aspects of prediction, responses to certain unpredictable ‘oddball’ stimuli can be found during REM (Atienza et al., 2000; Sculthorpe et al., 2009), but not Non-REM (Cote et al., 2001; Sabri and Campbell, 2005; see Ibáñez et al., 2009 for a review), implying that during REM sleep the brain is in a state conducive to the evaluation of predictive models.

**FIGURE 3:**
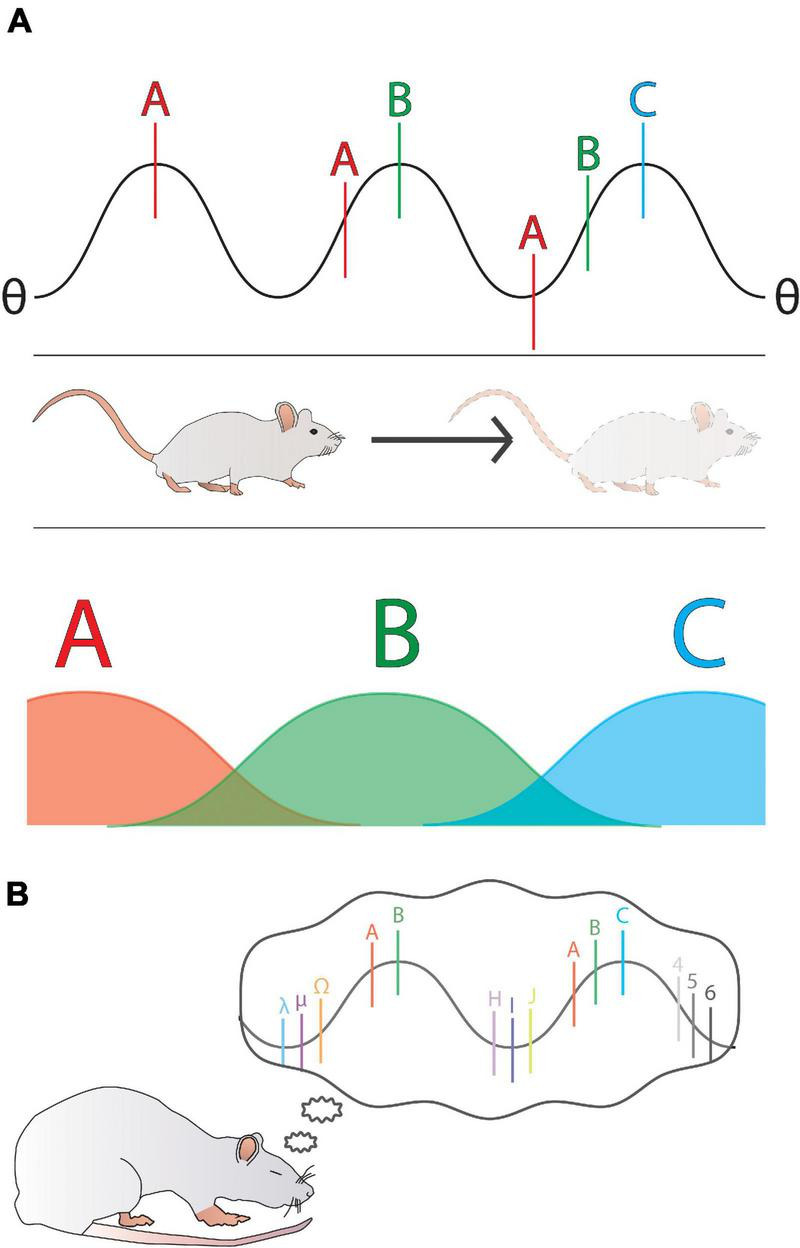
A general overview of phase precession as represented by an example of a rat moving through 1-dimensional space. **(A)** A hippocampal theta (θ) waveform has overlaid onto it the activity of three place cells (spiking frequencies are represented as A, B, and C; bottom). The position of each neuron’s spiking on the phase of theta is determined by the temporal sequence of the rat’s movement. As the rat moves through the regions represented by A, B, and C, the phase of each of these neuron’s spiking shifts further from 360°, such that each successive space is represented by an ordered sequence within a single theta cycle. **(B)** By reactivating theta during REM sleep, a rat replays the temporal sequences that became phase locked to theta during waking. It can be seen that the activity of many more neurons than A, B, and C could be encoded and linked onto theta, representing the role of theta in encoding more than just place fields and thus creating a variety of predictive models.

Some stronger evidence for a connection between predictions and REM sleep comes from ties between REM and activity in the hippocampus, a structure in mammalian brain associated with working memory (Tesche and Karhu, 2000). So-called ‘place cells’ in the hippocampus have been shown to encode specific and unique regions of physical space (O’Keefe and Dostrovsky, 1971), making them a prime candidate for processes involving consolidation of predictive models. Hippocampal replay of place cell firing sequences has been shown in rats (Lee and Wilson, 2002) and other animals (Ulanovsky and Moss, 2007) during both SWS and REM sleep. Interestingly, hippocampal replay is commonly associated with theta-band (4–8 Hz) activity during REM (Tesche and Karhu, 2000). As an endogenous rhythm, theta seems critical to the process of memory consolidation within the mammalian hippocampus (Cote et al., 2001), and ‘phase precession’ mechanisms (Figure 3) appear to be a key feature linking diverse firing sequences into a compact predictive code defined within different theta oscillation cycles (Jaramillo and Kempter, 2017). In essence, rather than being just a simple rate code, wherein different cells fire more when animals cross certain physical spaces (Figure 3A), each successive space is actually anticipated (due to past experience) as a unique firing sequence within a theta cycle (Jaramillo and Kempter, 2017). In this way, the hippocampus is able to encode information into the theta band activity at a timescale that is also conducive to spike timing dependent plasticity (STDP), meaning that confirmed predictions are strengthened and thus preserved as firing sequences (D’Albis et al., 2015), whereas prediction errors might jolt the system into a new coding sequence. It seems probable that a whole range of neuronal firing events are temporally organized within successive theta cycles, creating an opportunity for strengthening links among a variety of modalities and memories, not just sequential physical spaces. By reactivating theta (and thus, the predictive information provided by the aforementioned phase precession sequences) during REM sleep (Lee and Wilson, 2002; Figure 3B), the brain is able to effectively revisit these temporal sequences and regulate their synaptic strengths (Skaggs et al., 1996). It seems intuitive to extrapolate from this observation that such a role for REM sleep in optimizing predictions about physical navigation through space might generalize to other predictive capacities, such as sensorimotor or social.

In humans, Karni et al. (1994) showed that disruption of REM sleep impaired performance on tasks learned immediately prior to the REM deprivation but not on previously learned tasks, and when Non-REM sleep was disrupted there was no impairment to performance. Similar studies performed in mice have shown that the theta rhythm present during REM sleep is a critical component of this new-task consolidation (Boyce et al., 2016), probably through reactivation of neurons phase locked to the theta cycle. Given that theta is absent during SWS but present in wake and REM (Green and Arduini, 1954), it seems logical to infer that one aspect of REM may be engagement of wake-like processes to reorganize place cell activity and thereby allow the brain to build better predictive models. It is unknown, however, if invertebrates display predictive processes such as phase precession, but it is interesting to note that active sleep in *Drosophila* flies (Tainton-Heap et al., 2021) seems to be characterized by a theta-like (7–10 Hz) oscillation (Yap et al., 2017).

One potential clue that active sleep is associated with building predictive models comes from sleep ontogeny, or how sleep architecture changes through life. Most young animals need more sleep than adult animals (Kayser and Biron, 2016). In contrast, sleep is significantly reduced in old age, although this can be harder to disambiguate with encroaching neurodegenerative conditions such as Alzheimer’s and Parkinson’s, which are co-morbid with impaired sleep (Okawa et al., 1991). When partitioned between REM and Non-REM, it becomes clear that most of the change in sleep architecture through life (at least in humans) can be attributed to decreased REM, with this active sleep stage accounting for almost a third of a newborn’s life and only ∼5% of an elderly individual’s time, while Non-REM sleep duration stays comparatively more constant (Roffwarg et al., 1966). Intriguingly, REM sleep has been shown to occupy an even greater proportion of prenatal life, when infants are still developing in the womb (Peirano et al., 2003), with some proposing that early human brain development may be almost entirely REM-like (Coons and Guilleminault, 1982; Hobson and Friston, 2012). The observation that prenates and infants display substantially more REM sleep could suggest that this sleep stage has less to do with dreams *per se* (what might prenates dream about anyway?) and more to do with satisfying key needs of developing brains, such as neural reorganization (Cao et al., 2020). Following from our discussion above, one important need appears to be optimizing the capacity to make predictions about one’s actions, and thereby build models about one’s own body plan. Notably, human studies of proprioceptive efference copies have indicated a modulating role for theta oscillations (Stock et al., 2013), a role which would align well with the preponderance of REM in early brain development. It would seem reasonable to propose that most learning in the womb is proprioceptive, namely concerned with establishing control over different body parts and determining what sensory events have internal versus external causes. As newborns develop, other predictive models more relevant to life outside the womb need to be developed, and this ongoing need to learn, with perhaps a matching need for REM, continues through childhood but wanes in adulthood. Although not an explanation for REM sleep, this correlation with sleep ontogeny provides a powerful entry point into potentially exploring active sleep in non-human animals, from other mammals to invertebrates. This is because such an explanation sidesteps any need to explain dreaming (what does it matter what prenates – or flies – might be dreaming about?) and focusses instead on functional explanations linked to optimizing predictive models – something highly relevant to most motile creatures that have to anticipate the consequences of their actions.

## Discussion

The evolution of sleep and attention is probably intertwined (Kirszenblat and van Swinderen, 2015), and here we propose that it is active sleep specifically that has co-evolved with animals’ capacity to pay attention to surprising events in their environment. Whereas quiet sleep (or SWS in mammals and birds) is increasingly found to be associated with homeostatic repair processes that collectively appear to be attempts at reducing cellular entropy in the brain following waking activity, active sleep may instead reflect cognitive homeostatic process aimed at optimizing how animals predict the world. This hypothesis has interesting implications for the evolution of subjective awareness across animals, and for the role of active sleep in curating this capacity throughout the life of individual animals. Specifically, we propose that the ongoing debate on the origins of consciousness in animals (Barron and Klein, 2016) could be productively informed by understanding which animals have evolved a need for active sleep alongside quiet sleep.

Brains could be viewed as evolving prediction machines. We discussed earlier how emotional responses associated with prediction errors might be important for forming new memories, to enable brains to become even better prediction machines. Thus, a joke is typically funny the first time because of some unexpected twist, but rarely funny the second time: we predict the twist. Other than humans, animals don’t seem to joke much, but most animals are probably well tuned to detect prediction errors more relevant to their individual niches. Most animals might make use of endogenous arousal and valence systems to detect prediction errors, and thereby highlight the need for an updated prediction. Yet, this process needs to be finely tuned. Too many prediction errors might indicate a maladaptive inability to generalize, while too few prediction errors might result in an inability to learn anything new. Herein lies a paradox: the mechanism that brains seem to employ to detect and correct prediction errors (emotion, or arousal) is the same quality that brains are attempting to eliminate by becoming better prediction machines.

Indeed, this paradox has been discussed in machine learning and philosophy. For example, regarding the difference between novelty and surprise in computational neuroscience (Barto et al., 2013; Schwartenbeck et al., 2013), or in the ‘dark room’ problem in philosophy (Friston et al., 2012), which puts forward the following conundrum: if brains are designed to minimize surprise, then why don’t animals act to minimize unpredictable events by seeking environments that remove certain stimuli entirely (e.g., a dark room)? A resolution to this paradox has been proposed at the level of predictive coding theory: the minimization of prediction errors in the moment could be viewed as fundamentally different from choosing actions that will minimize prediction errors in the future. Technically, prediction errors correspond to surprise, while ‘expected’ prediction errors – consequent on action – correspond to uncertainty. This follows because surprise is self-information in information theory and expected surprise is entropy or uncertainty. Thus, there is a key difference between a surprising event that was unpredicted and choosing an action that you expect to bring about unpredictable outcomes. Minimizing expected surprise is the tenet of active inference and rests upon a good generative or internal model of the consequences of actions. A role for sleep in this setting has been proposed previously (e.g., see Friston et al., 2017). Active sleep could provide an opportunity for the brain to simulate and test a broad range of internal models, which is probably a more adaptive strategy than seeking a metaphorical dark room of zero surprises.

Imagine a brain becoming so good at predicting everything in its environment that it never becomes surprised anymore, and thus never evokes an emotional response to highlight a prediction error. Such a brain might not be very different from a computer: just an input/output system working within an invariant universe. Such a brain would not need emotion, since in a world of perfect predictability there is no surprise and thus no need to consolidate new memories. Indeed, it might be doubtful whether such a brain would be conscious, in the way that term pertains to subjective experience (Barron and Klein, 2016). A brain moving toward zero surprises might sound adaptive, but it probably isn’t. This is because the world is never entirely predictable. A brain in a closed environment (e.g., a baby in the womb, a monk in a monastery, or a fly in a bottle) may achieve close to perfect predictability in that specific environment, but this does not do it any good outside that environment. We are always surprised, because our world is always changing, and this requires continuously updating our models of the world. This is important from the point of view of cognitive flexibility and adaptability.

Cognitive flexibility comes hand-in-hand with minimizing redundancy and maintaining a degree of latitude when forming accurate accounts of the (waking) sensorium. One view of active sleep that speaks to this imperative builds on ideas from statistics and machine learning (Hinton et al., 1995). In this setting, the maximization of model evidence entails a minimization of statistical complexity. This can be seen from many perspectives. For example, in the free energy principle proposed by Friston et al. (2006), the implicit maximization of entropy is one way of ensuring that we keep our options open when forming beliefs about states of affairs in the world (Hobson and Friston, 2012). This may seem in opposition to proposed deep sleep functions, which are aimed at decreasing entropy or complexity in the brain, which has been formulated in the context of minimizing synaptic connections (Tononi and Cirelli, 2006). It is possible that active sleep – and the rehearsal of narratives and contingencies accumulated during the day – is similarly in the service of removing redundant connections and thereby minimizing complexity. Cognitive flexibility could thus be seen as emanating from processes that preclude over-fitting overly parametrized internal models (with redundant and exuberant synaptic connections) (Hoel, 2021). This view would tie neatly with the synaptic homeostasis hypothesis that has been attributed to deep sleep in higher animals (Huber et al., 2004; Tononi and Cirelli, 2006).

A related view however might be that the neural reorganization that seems inherent to active sleep (Cao et al., 2020) ensures that the crucial cellular repair/homeostatic processes engaged during deep (quiet) sleep do not compromise cognitive flexibility. Thus, what begins as a necessary model-building exercise during brain development persists throughout life (albeit often to a lesser degree; Herman et al., 1991; Hobson, 2009a), as a crucial mechanism for maintaining cognitive flexibility. By drawing links among events (or neuronal groups) which would not ordinarily be associated in waking life, active sleep might ensure that valence systems (how value is assigned) remain tuned at an optimal level, allowing for an appropriate level of surprise while awake. One way to do this may be to disconnect the waking brain from the outside world for extended periods of time. In this sense, a key function of active sleep – in any animal – may be to entertain a quasi-infinite range of alternate possibilities (by replaying or remixing neural sequences, as in Figure 3B), to ensure the waking brain remains just enough surprised about the real world to keep paying attention and learning. Consciousness is thus adaptive, but it doesn’t come for free. We need to dream to keep from becoming habit-driven, entropy-minimizing robots.

While the link between attention and consciousness remains debated (e.g., see De Brigard and Prinz, 2010; van Boxtel et al., 2010), a focus on optimizing prediction provides an effective strategy to investigate a role for active sleep in simpler animal models such as flies. In predictive processing and active inference, attention is usually described as assigning greater precision to certain sensory streams or posterior beliefs (Feldman and Friston, 2010). Simply put, precision in this instance is an estimate of predictability. Physiologically, it is thought to be encoded by neuromodulatory mechanisms that control synaptic gain (Kanai et al., 2015). Thus, assigning precision in a context-sensitive fashion (i.e., cognitive flexibility) looks very much like attention. The key point here is that exactly the same neuromodulatory mechanisms that underwrite attention – and the deployment of precision during hierarchical predictive processing – are those thought to be responsible for active sleep and dreaming (Hobson, 2009b). This speaks to our notion that dreaming and attention may inherit from the same (classical) neuromodulatory systems.

The idea that dreaming might shape our consciousness is not new (Hobson, 2009a; Hobson et al., 2021; Windt, 2021). What is new is the realization that many other animals, including even flies, seem to have an active sleep stage. This suggests that something more primordial than consciousness is being attended to by periodically uncoupling a waking brain from the outside world. This view implies that this primordial quality is adaptive, meaning that it helps animals survive. This view also suggests that this trait might be a feature of all animals that show any evidence of active sleep. We propose that what is being curated here is a balance between prediction and surprise, which shapes how animals pay attention. Rather than being a simple indicator for which animals are conscious and which are not, we propose this as an effective strategy to understand how subjective awareness may have evolved from such a mechanism. It will for example be interesting to verify the extent of active sleep across the animal kingdom and see how this might correlate with different animals’ capacity to optimize prediction error signals.

## Data Availability Statement

The original contributions presented in the study are included in the article/supplementary material, further inquiries can be directed to the corresponding author.

## Author Contributions

MVDP and BVS wrote the manuscript. Both authors contributed to the article and approved the submitted version.

## Conflict of Interest

The authors declare that the research was conducted in the absence of any commercial or financial relationships that could be construed as a potential conflict of interest.

## Publisher’s Note

All claims expressed in this article are solely those of the authors and do not necessarily represent those of their affiliated organizations, or those of the publisher, the editors and the reviewers. Any product that may be evaluated in this article, or claim that may be made by its manufacturer, is not guaranteed or endorsed by the publisher.
